# Sustained response to atogepant in episodic migraine: post hoc analyses of a 12-week randomized trial and a 52-week long-term safety trial

**DOI:** 10.1186/s10194-024-01783-6

**Published:** 2024-05-21

**Authors:** Richard B. Lipton, Stephanie J. Nahas, Patricia Pozo-Rosich, Tanya Bilchik, Peter McAllister, Michelle Finnegan, Yingyi Liu, Natty Chalermpalanupap, Brett Dabruzzo, David W. Dodick

**Affiliations:** 1 Albert Einstein College of Medicine, Bronx, NY USA; 2 Thomas Jefferson University, Philadelphia, PA USA; 3 Headache Unit, Department of Neurology, Vall d’Hebron University Hospital, Barcelona, Spain; 4 Headache Research Group, Vall d’Hebron Institute of Research, Universitat Autònoma de Barcelona, Barcelona, Spain; 5 Department of Neurology, Yale University School of Medicine, New Haven, CT USA; 6 New England Institute for Neurology & Headache, Stamford, CT USA; 7 AbbVie, North Chicago, IL USA; 8 Mayo Clinic, Phoenix, AZ USA; 9 Atria Academy of Science and Medicine, New York, NY USA

**Keywords:** CGRP, Responders, Migraine

## Abstract

**Background:**

Atogepant is an oral calcitonin gene–related peptide receptor antagonist approved for the preventive treatment of migraine in adults. These analyses evaluated the proportions of clinical trial participants who experienced sustained responses to atogepant over 12 or 52 weeks of treatment.

**Methods:**

These were post hoc analyses of ADVANCE, a 12-week, double-blind, randomized trial of atogepant 10, 30, and 60 mg once daily vs. placebo for the preventive treatment of episodic migraine, and a separate open-label long-term safety (LTS) trial of atogepant 60 mg once daily over 52 weeks. The 60 mg dose of atogepant was used to detect safety issues. An initial response was defined as ≥50%, ≥75%, or 100% reduction from baseline in MMDs in month 1 for ADVANCE or quarter 1 for the LTS trial. The proportions of participants who continued to experience a response above each response-defining threshold through each subsequent month (for ADVANCE) or each quarter (for LTS) were calculated.

**Results:**

In ADVANCE, sustained response rates during months 2 and 3 varied with dose and were as follows: 70.8–81.1% following an initial ≥50% response, 47.3–61.9% following an initial ≥75% response, and 34.8–41.7% following an initial 100% response. Of those who experienced an initial ≥75% or 100% response during month 1, more than 79% continued to experience at least a 50% response during both months 2 and 3. During the LTS trial, sustained response rates through quarters 2, 3, and 4 were 84.7% following an initial ≥50% response, 72.6% following an initial ≥75% response, and 37.8% following an initial 100% response. Of those who experienced an initial ≥75% or 100% response during quarter 1, more than 90% continued to experience at least a 50% response through quarters 2, 3, and 4.

**Conclusion:**

Over 70% of participants who experienced an initial response with atogepant treatment had a sustained response with continued treatment.

**Trial registration:**

ClinicalTrials.gov: NCT03777059 (submitted: December 13, 2018); NCT03700320 (submitted: September 25, 2018).

## Introduction

Migraine is a highly prevalent neurologic disease worldwide that is characterized by recurrent attacks of headache pain, often accompanied by phonophobia, photophobia, and nausea [1]. Migraine causes more years lived with disability than all other neurologic diseases combined and is the leading cause of disability in young adult women [2–4]. People with migraine report nearly double the rates of absenteeism, presenteeism, and overall work impairment compared with those without migraine, and approximately one third of people with migraine report a negative impact on their career and financial worry as a result of the disease [5, 6]. The Migraine in America Symptoms and Treatment (MAST) study found that people with migraine were upwards of three times more likely to suffer from insomnia, depression, anxiety, and gastric ulcer/gastrointestinal bleeding compared with those without migraine [7, 8].

Although migraine is common, it tends to be underdiagnosed and undertreated [9]. Many people with episodic migraine (EM) are highly disabled by the disease and are in need of preventive treatment [10]. Despite the high disability associated with migraine, only a minority (∼ 10–20%) of people with migraine who are eligible for preventive treatment based on the 2021 American Headache Society consensus statement algorithm are currently taking a migraine preventive medication [11]. Ideally, preventive treatments for migraine should have both a rapid onset of action and sustained efficacy. However, current conventional oral preventive treatments require titration and may take weeks or months to demonstrate maximum efficacy.

Historically, oral medications for the preventive treatment of migraine tend to have low rates of adherence and persistence [12, 13]. Often, the reasons for poor adherence or discontinuation of oral preventive treatment include insufficient efficacy, insufficient tolerability, or both [13–15]. Results from the second International Burden of Migraine Study, a web-based, cross-sectional survey, revealed that 36.8–47.6% of respondents with EM discontinued their oral preventive treatment due to lack of efficacy, and 34.8–49.0% discontinued because of side effects [14]. In a cross-sectional study of disease burden and treatment patterns among people with migraine, over 70% of respondents with EM who reported switching or discontinuing preventive treatment indicated lack of efficacy or safety/tolerability as the reason [15]. These data suggest that both rapid onset and sustained efficacy are important attributes of migraine preventive treatments.

Atogepant is an oral calcitonin gene–related peptide (CGRP) receptor antagonist approved for the preventive treatment of migraine (EM and chronic migraine [CM]) in adults [16]. The ADVANCE trial was a pivotal 12-week phase 3 trial that demonstrated the efficacy and safety of atogepant 10, 30, and 60 mg once daily for the preventive treatment of EM [17]. In order to evaluate the safety and tolerability of atogepant for the preventive treatment of EM over a longer period of time, a separate open-label, long-term safety (LTS) trial among participants who had either previously completed a phase 2b/3 atogepant trial or were naive to atogepant was conducted, evaluating the maximum dose (60 mg once daily) over 52 weeks [18].

In multiple clinical trials of atogepant, favorable tolerability and adequate efficacy have been demonstrated [17–20]. In the extension trial of ADVANCE, the tolerability of atogepant was demonstrated with more than 70% of the safety population continuing atogepant for at least 9 months [20]. Moreover, rates of discontinuation of atogepant due to adverse events were only 2.7% for the 60 mg dose in the ADVANCE trial [17]. Furthermore, efficacy was also favorable in both the ADVANCE and LTS trials. Over 60% of participants treated with atogepant 60 mg experienced a 50% or greater reduction from baseline in monthly migraine days (MMDs) over 12 weeks [17, 18]. This endpoint is considered an important clinical outcome, as a 50% reduction in MMDs is aligned with clinical trial guidelines for the development of preventive treatments for migraine [21].

Given that migraine is a chronic disease, it is imperative that preventive treatments for migraine provide continued efficacy with long-term benefits. In an interim analysis of data from the Chronic Migraine Epidemiology and Outcomes–International study, over one-third of respondents in the United States who discontinued a preventive medication reported insufficient efficacy as a factor contributing to their decision to discontinue [22]. While the safety and efficacy of atogepant have been previously established, the degree to which treatment response is sustained in people who report an initial response has not been evaluated. Sustained responses, based on thresholds of prespecified reductions in MMDs over a given period of time, allow for the evaluation of long-term efficacy. The objective of these post hoc analyses was to assess the proportions of participants who experienced sustained responses of ≥50%, ≥75%, or 100% reduction in MMDs over 12 and 52 weeks of atogepant treatment.

## Methods

### Study design

Full study details of the ADVANCE and LTS trials have previously been published [17, 18]. Briefly, the ADVANCE trial was a 12-week, double-blind, placebo-controlled, phase 3 trial conducted in the United States from December 14, 2018, to June 19, 2020, that evaluated the safety and efficacy of atogepant for the preventive treatment of EM. Participants were randomized (1:1:1:1) to treatment with once-daily atogepant 10 mg, 30 mg, or 60 mg, or placebo. The LTS trial was a multicenter, randomized, open-label trial conducted in the United States from October 8, 2018, to May 29, 2020, that evaluated the safety and tolerability of atogepant for the preventive treatment of EM for up to 52 weeks. Eligible participants included those who had completed the phase 2b/3 atogepant trial and a group of atogepant-naive participants. Participants from the phase 2b/3 trial had a minimum of a 6-month gap from the end of that trial to enrollment in the LTS study. At the time of enrollment, eligible participants met criteria for migraine according to the International Classification of Headache Disorders, 3rd edition, had 4 to 14 migraine days per month, and completed a pretreatment diary to establish MMDs at baseline. For the LTS study, participants were randomized (5:2) to treatment with once-daily atogepant 60 mg or standard care (SC). Participants randomized to the SC arm received a physician-selected oral migraine preventive medication (e.g., topiramate, amitriptyline, propranolol) as their initial treatment. Permitted preventive medications in the SC arm were those recognized as safe and effective for the preventive treatment of migraine, based on investigator’s judgment, and full details have been previously described [18]. The SC arm was included to contextualize the long-term safety of atogepant by providing comparative data in a manner consistent with clinical practice; efficacy data were not collected in the SC arm. Post hoc analyses of ADVANCE and LTS trials were performed to assess sustained response associated with atogepant treatment. Data from ADVANCE and the LTS trial were analyzed separately.

The ADVANCE and LTS trials were approved by an institutional review board or independent ethics committee and were registered at ClinicalTrials.gov NCT03777059 (ADVANCE) and NCT03700320 (LTS trial). For each trial, participants provided written informed consent. All study conduct was in accordance with the International Conference on Harmonisation Guidelines for Good Clinical Practice.

### Participants

Inclusion and exclusion criteria for each trial were published previously [17, 18]. Briefly, eligible participants were adults with 4 to 14 migraine days per month in the 3 months before visit 1 and in the 28-day baseline period. Participants were diagnosed with migraine with or without aura according to the International Classification of Headache Disorders, 3rd edition [1], for a minimum of 1 year with the diagnosis made prior to the age of 50 years.

### Outcomes

For these post hoc analyses, participants were categorized as having an initial response if they experienced ≥50% reduction from baseline in MMDs in month 1 (4 weeks after starting treatment) for ADVANCE or in quarter 1 (first 12 weeks) for the LTS trial. Participants who experienced this initial ≥50% response were further categorized using higher response thresholds of ≥75%, or 100%. The proportions of participants experiencing either the same initial response or ≥50% response through each subsequent month for ADVANCE or through each quarter for the LTS study were calculated. Missing data were treated conservatively. If a participant with an initial response had missing data for some timepoint (month/quarter) later in the trial, and they did not experience response at the nonmissing timepoint, they were included in the analyses as not experiencing a sustained response. If they did experience the response in the nonmissing timepoint, but had missing data for other timepoints, they were excluded from the analyses.

For ADVANCE, the proportions of participants who experienced a <50% response in month 1 but proceeded to experience a ≥50% response in subsequent months were calculated.

### Statistical analysis

Response rates were previously presented in the overall modified intent-to-treat (mITT) population for both trials [17, 18]. These post hoc analyses focused on the subgroups of participants with an initial response in each trial and evaluated the proportions of those who continued to experience a given response threshold (e.g., ≥50%, ≥75%, or 100% reduction in MMDs from baseline) during the remainder of the 12- or 52-week trial. An initial response was defined as a ≥50%, ≥75%, or 100% reduction in MMDs from baseline during the first month of treatment in ADVANCE or during the first quarter of open-label treatment in the 52-week LTS trial. For ADVANCE, the subgroup of participants with a < 50% reduction in MMDs from baseline in month 1 who proceeded to experience a ≥50% reduction in MMDs from baseline in month 2 and month 2 or 3 was assessed. These analyses used observed data for all participants at each assessment period. A month was defined as each 4-week treatment interval. Data for each quarter of the LTS trial were based on the monthly average of MMDs (3 months for quarters 1–3, 4 months for quarter 4).

## Results

### Participants

Of 659 participants included in the mITT population in the ADVANCE trial, 214 participants received atogepant 10 mg, 223 participants received atogepant 30 mg, and 222 participants received atogepant 60 mg (Table 1). Mean MMDs at baseline were similar across dose groups, ranging from 7.5 to 7.9 days. In month 1 of the ADVANCE trial, a ≥50% initial MMD response was experienced by 48.9% (109/223; 30 mg) to 61.1% (135/221; 60 mg) of participants, a ≥75% initial response was experienced by 26.9% (60/223; 30 mg) to 39.4% (87/221; 60 mg) of participants, and a 100% initial response was experienced by 11.7% (26/223; 30 mg) to 19.0% (42/221; 60 mg) of participants.

**Table 1:** Initial response subgroups and baseline monthly migraine days

	ADVANCE			LTS Trial
	10 mg	30 mg	60 mg	60 mg
**N (mITT population)**	214	223	222	521
**Responses during initial period**,^**a**^** n/N1 (%)**				
≥50% response subgroup	105/213 (49.3)	109/223 (48.9)	135/221 (61.1)	325/521 (62.4)
≥75% response subgroup	58/213 (27.2)	60/223 (26.9)	87/221 (39.4)	198/521 (38.0)
100% response subgroup	30/213 (14.1)	26/223 (11.7)	42/221 (19.0)	55/521 (10.6)
**MMDs at baseline, mean (SD)**	7.5 (2.5)	7.9 (2.3)	7.8 (2.3)	7.3 (2.6)

LTS, long-term safety; mITT, modified intent-to-treat; MMD, monthly migraine day

^a^Initial period defined as 4 weeks for ADVANCE and 12 weeks for the LTS trial

In the LTS trial, 744 participants underwent randomization. Of these, 14.4% had participated in the phase 2b/3 atogepant trial and 85.6% had not previously taken atogepant. A total of 521 participants were included in the mITT population and received atogepant 60 mg. Participants reported a mean of 7.3 MMDs. Of these, 62.4% (325/521) experienced a ≥50% initial response in the first quarter, 38.0% (198/521) experienced a ≥75% initial response in the first quarter, and 10.6% (55/521) experienced a 100% initial response in the first quarter.

### Efficacy results

#### ADVANCE

**Fig. 1:**
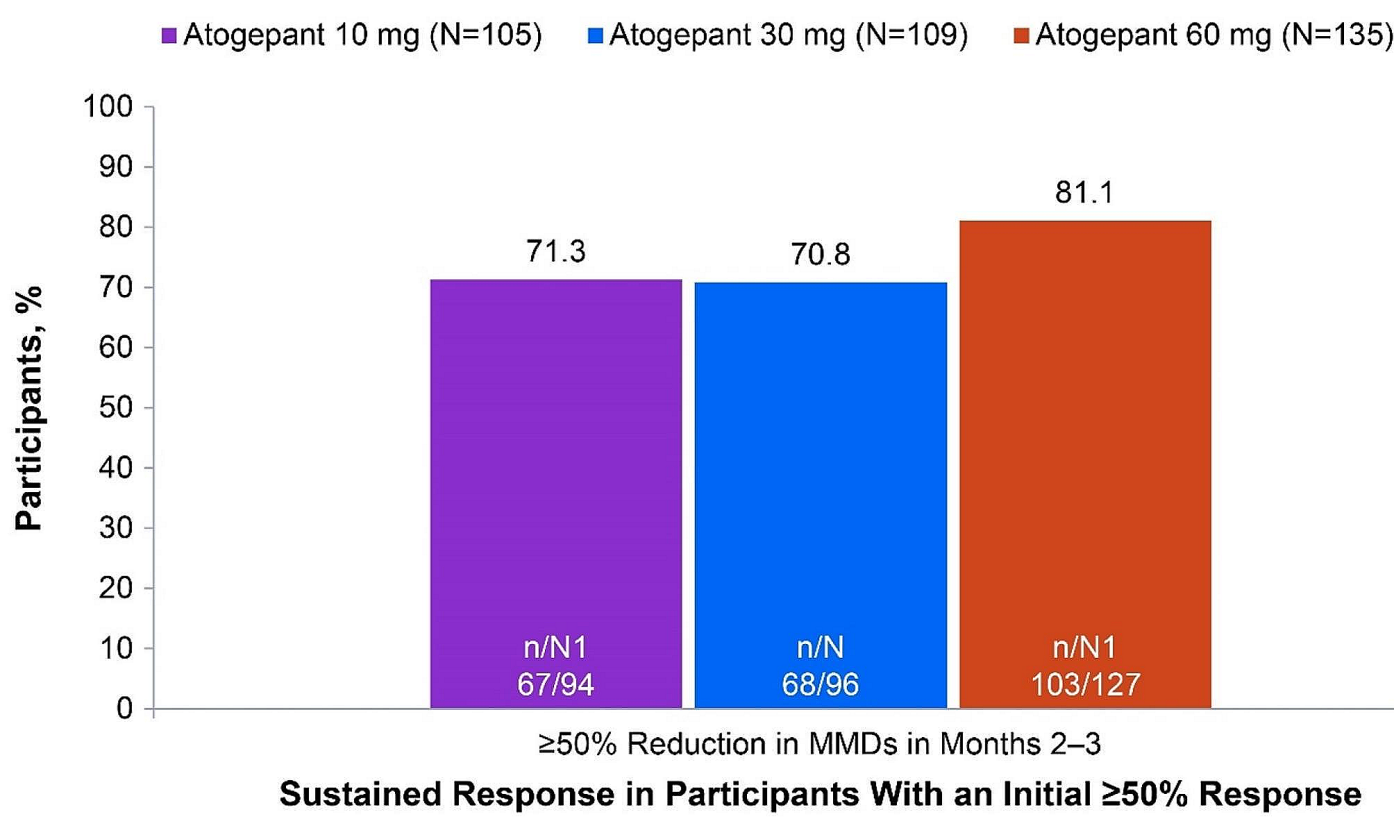
Sustained Response in Both Months 2 and 3 Among Participants With a ≥50% Response in Month 1 (ADVANCE). MMDs, monthly migraine days; N, participants with a ≥50% reduction in MMDs in month 1; n, number of participants within a specific category; N1, number of participants available for analysis

**Fig. 2:**
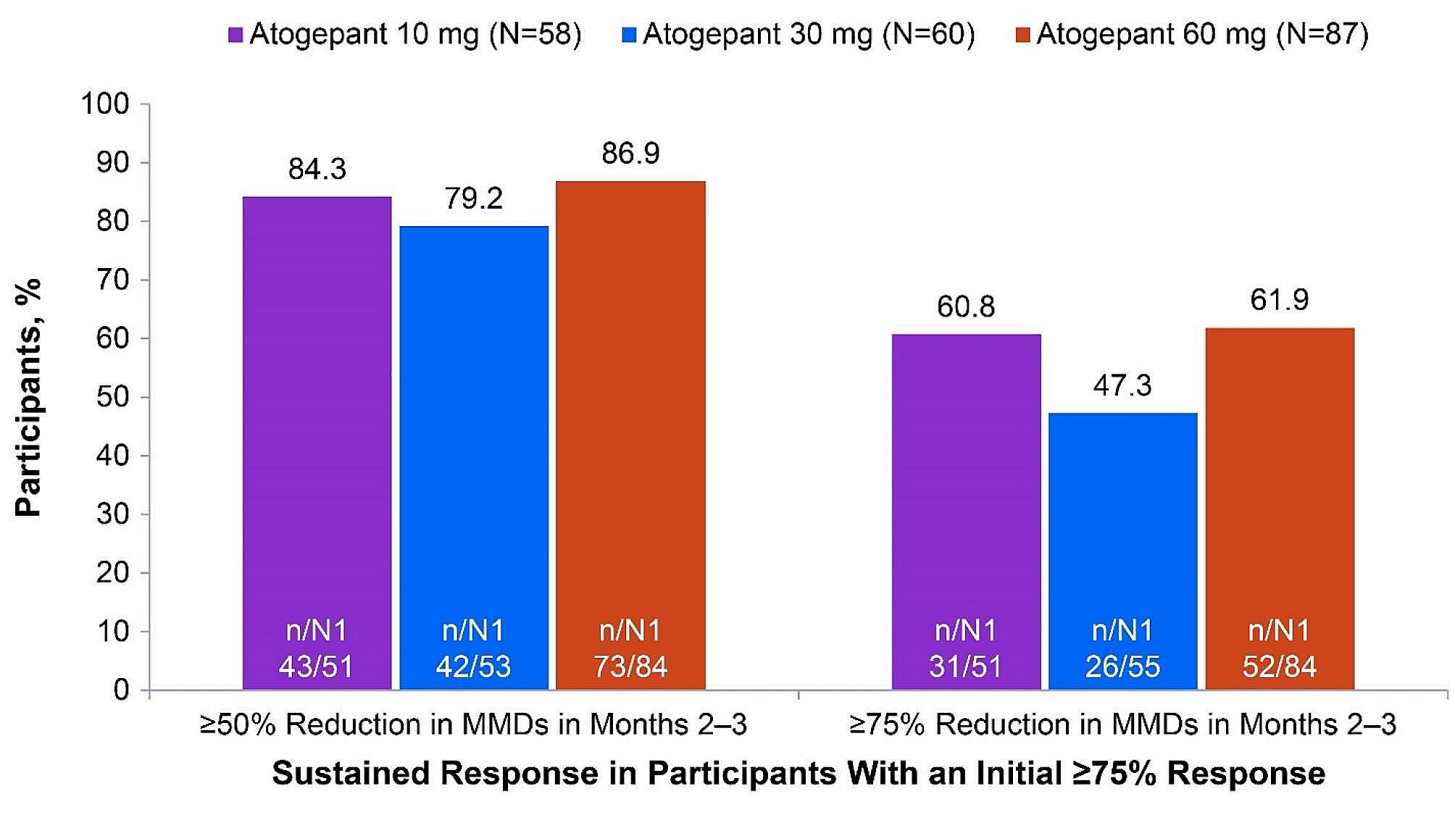
Sustained Response of ≥50% in Both Months 2 and 3 Among Participants With a ≥75% Response in Month 1 (ADVANCE). MMDs, monthly migraine days; N, participants with a ≥75% reduction in MMDs in month 1; n, number of participants within a specific category; N1, number of participants available for analysis

**Fig. 3:**
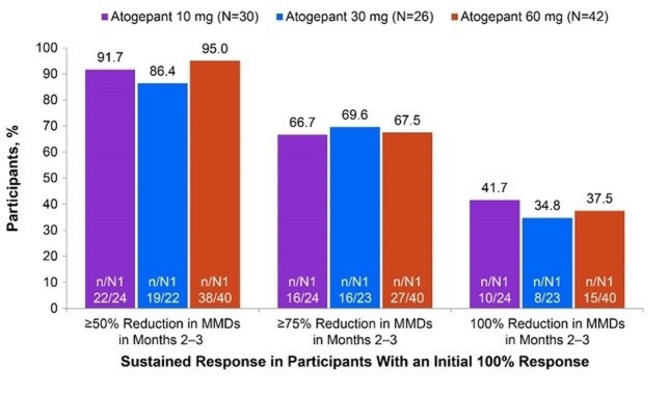
Sustained Response of ≥50% in Both Months 2 and 3 Among Participants With a 100% Response in Month 1 (ADVANCE). MMDs, monthly migraine days; N, participants with a 100% reduction in MMDs in month 1; n, number of participants within a specific category; N1, number of participants available for analysis

Of ADVANCE participants who experienced a ≥50% response within month 1, 70.8% (68/96; 30 mg) to 81.1% (103/127; 60 mg) continued to experience this initial ≥50% response through the remainder of the 12-week double-blind treatment period (months 2 and 3) (Fig. 1). Among those with an initial ≥75% response, 47.3% (26/55; 30 mg) to 61.9% (52/84; 60 mg) continued to experience a ≥75% response through months 2 and 3, and 79.2% (42/53; 30 mg) to 86.9% (73/84; 60 mg) had a ≥50% response through months 2 and 3 (Fig. 2). Among those with an initial 100% response, 34.8% (8/23; 30 mg) to 41.7% (10/24; 10 mg) continued to experience a 100% response, 66.7% (16/24; 10 mg) to 69.6% (16/23; 30 mg) had a ≥75% response, and 86.4% (19/22; 30 mg) to 95.0% (38/40; 60 mg) had a ≥50% response through months 2 and 3 (Fig. 3).

**Fig. 4:**
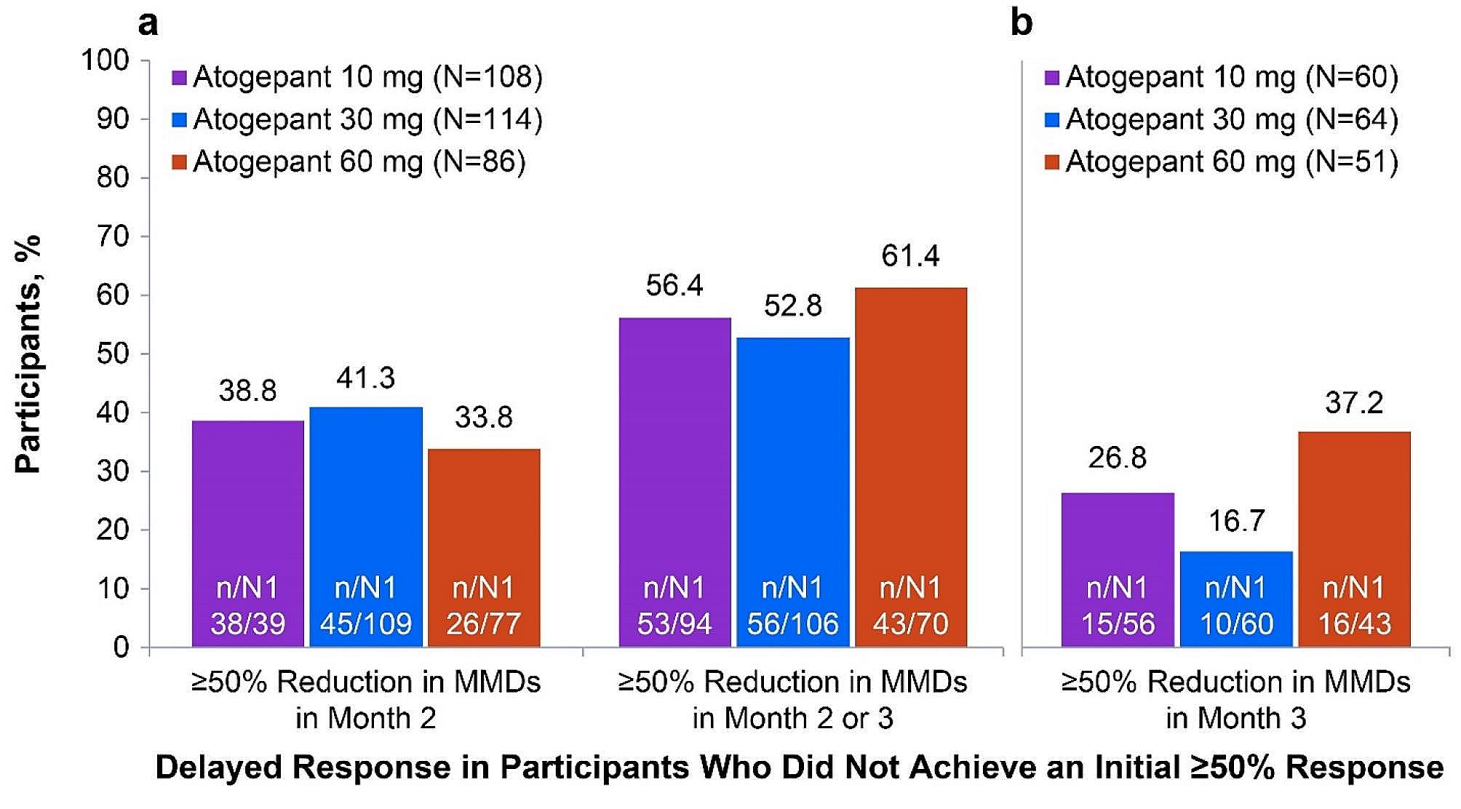
Delayed Response of ≥50% Among Participants Without a ≥50% Response in Month 1 (**a**) and Months 1 and 2 (**b**) (ADVANCE). MMDs, monthly migraine days; N, total number of participants included in the modified intent-to-treat population; n, number of participants not achieving response; N1, total number of participants with determinable data

Of ADVANCE participants who did not experience a ≥50% response within month 1, 33.8% (26/77; 60 mg) to 41.3% (45/109; 30 mg) had ≥50% reduction from baseline in MMDs in month 2 and 52.8% (56/106; 30 mg) to 61.4% (43/70; 60 mg) experienced ≥50% reduction from baseline in MMDs in either month 2 or 3 (Fig. 4A). Among participants who did not have a ≥50% reduction in MMDs in month 1 or 2, 16.7% (10/60; 30 mg) to 37.2% (16/43; 60 mg) experienced a ≥50% reduction in MMDs in month 3 (Fig. 4B).

#### LTS trial

**Fig. 5:**
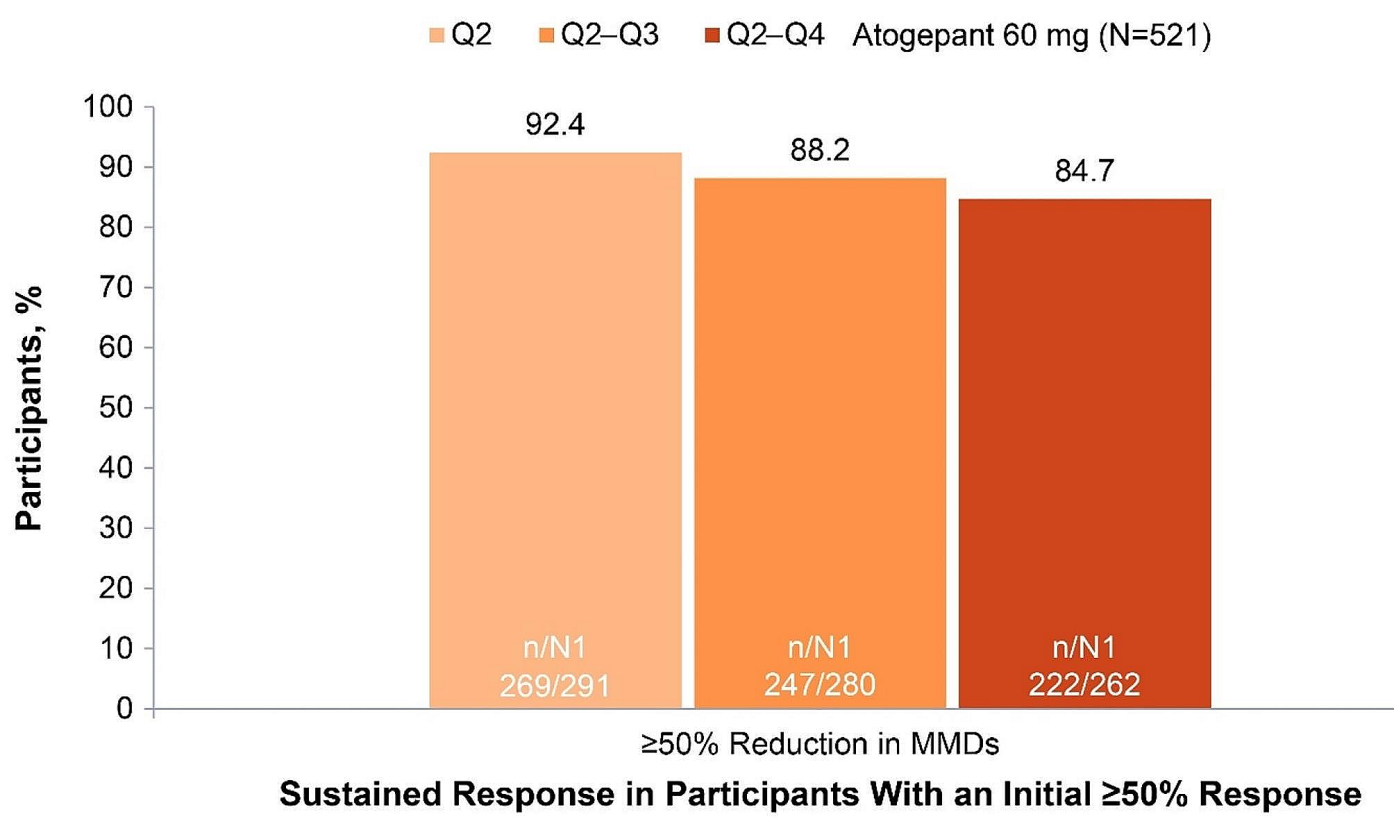
Sustained Response Over 52 Weeks Among Participants With a ≥50% Response in Q1 (52-Week Trial). MMDs, monthly migraine days; N, participants included in the modified intent-to-treat population; n, number of Q1 with initial response who had sustained response in Q2, Q2–Q3, or Q2–Q4; N1, number of Q1 with initial response from participants with determinable data at Q2, Q2–Q3, or Q2–Q4; Q, quarter

**Fig. 6:**
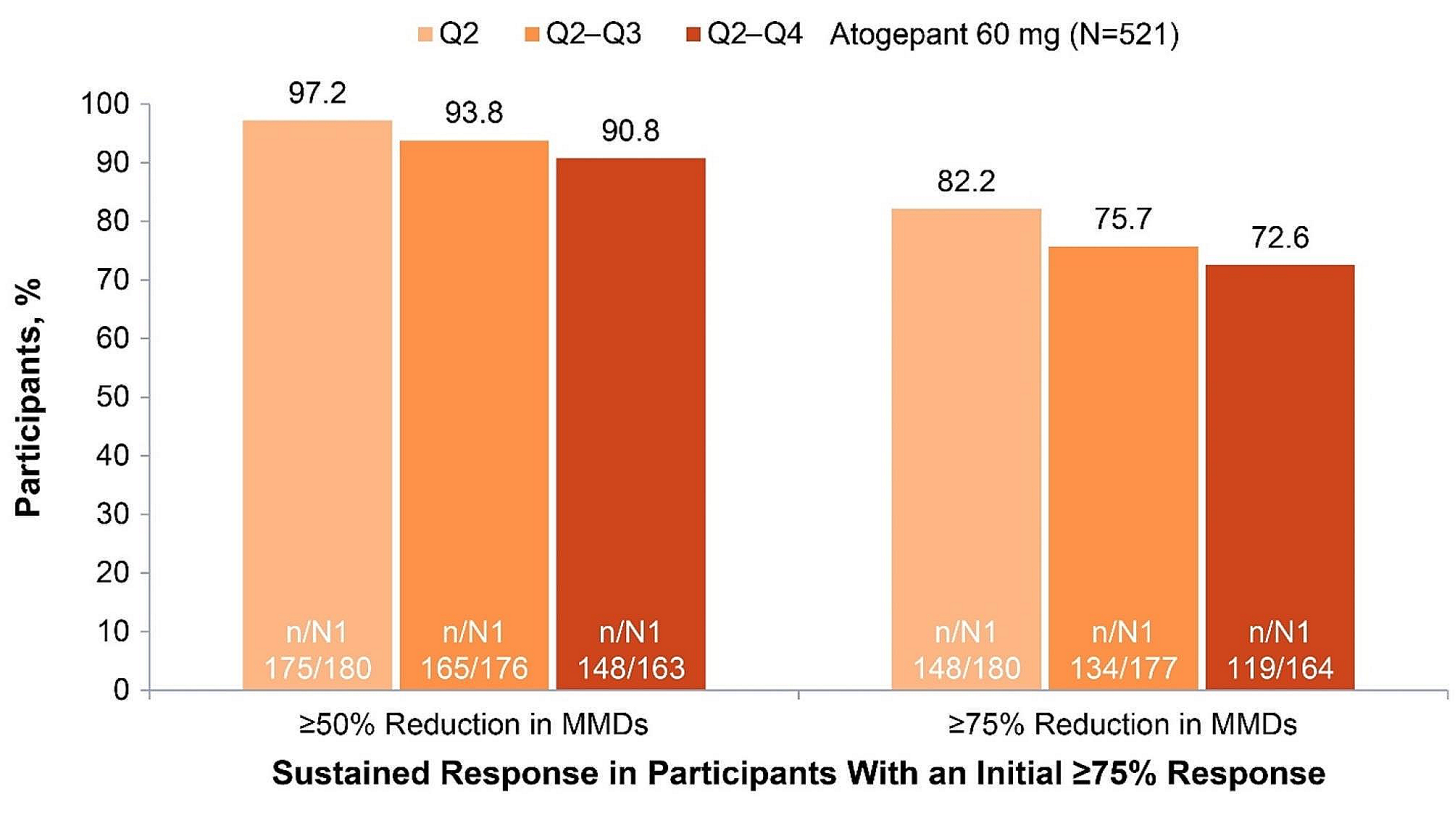
Sustained Response of ≥50% Over 52 Weeks Among Participants With a ≥75% Response in Q1 (52-Week Trial). MMDs, monthly migraine days; N, participants included in the modified intent-to-treat population; n, number of Q1 with initial response who had sustained response in Q2, Q2–Q3, or Q2–Q4; N1, number of Q1 with initial response from participants with determinable data at Q2, Q2–Q3, or Q2–Q4; Q, quarter

**Fig. 7:**
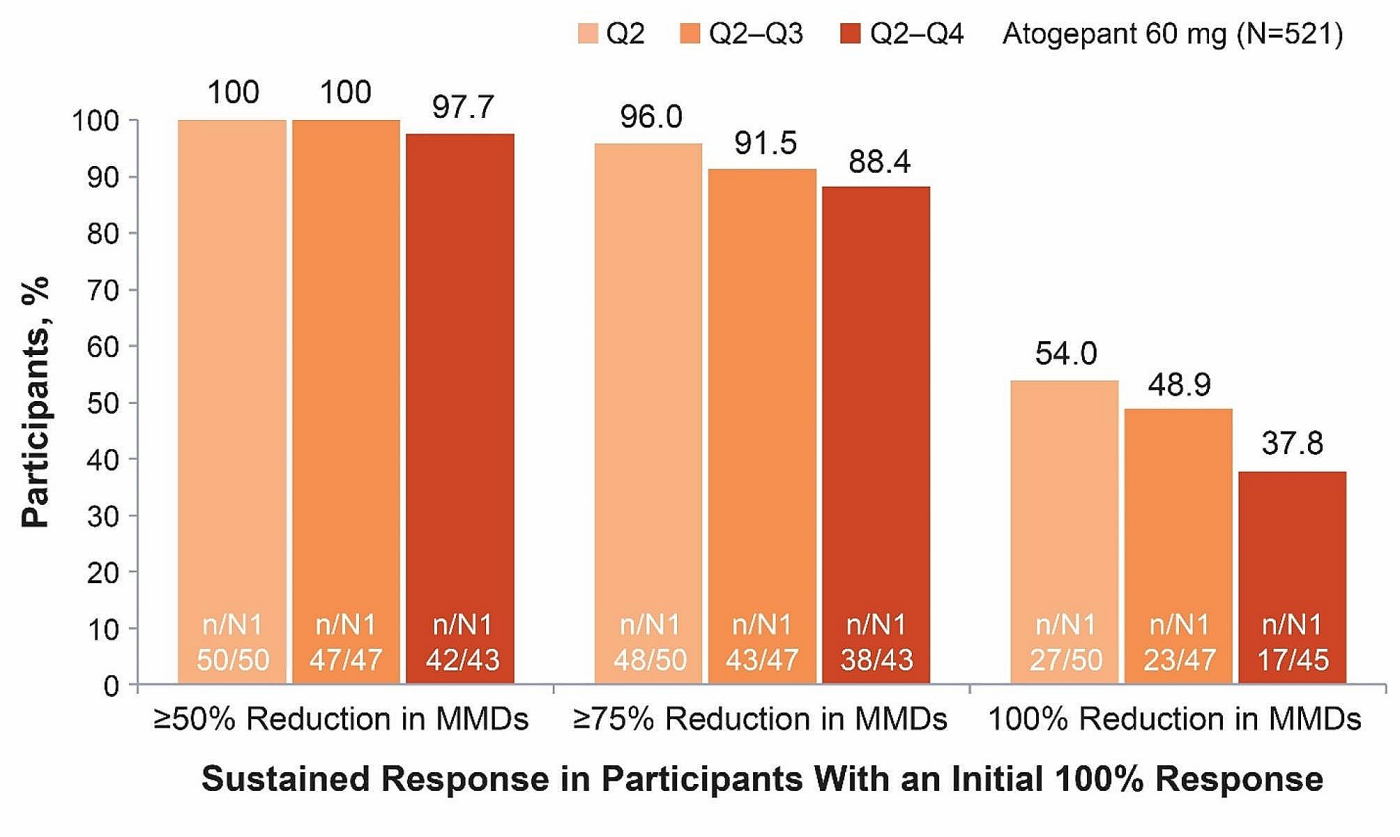
Sustained Response of ≥50% Over 52 Weeks Among Participants With a 100% Response in Q1 (52-Week Trial). MMDs, monthly migraine days; N, participants included in the modified intent-to-treat population; n, number of Q1 with initial response who had sustained response in Q2, Q2–Q3, or Q2–Q4; N1, number of Q1 with initial response from participants with determinable data at Q2, Q2–Q3, or Q2–Q4; Q, quarter

In the 52-week LTS trial, 84.7% (222/262) of participants treated with atogepant 60 mg who had an initial ≥50% response experienced sustained response throughout all subsequent quarters (quarters 2–4) (Fig. 5). Among those with an initial ≥75% response, 72.6% (119/164) continued to experience the same ≥75% response throughout the 52-week trial, and 90.8% (148/163) had a ≥50% response in all subsequent quarters (Fig. 6). Among those with an initial 100% response, 37.8% (17/45) continued to experience a 100% response throughout each subsequent quarter of the 52-week trial; 88.4% (38/43) had a ≥75% response, and 97.7% (42/43) had a ≥50% response in all subsequent quarters (Fig. 7).

## Discussion

The 12-week results of the ADVANCE trial and the 52-week results of the LTS trial indicate that high proportions of participants with EM experience a meaningful reduction of ≥50% in MMDs in the first 4 weeks, and that those responses are usually sustained. In both ADVANCE and the LTS trial, most participants who had a ≥50% initial response continued to experience the response throughout their treatment period. For the 60 mg dose in the ADVANCE trial, 61.1% had at least a 50% reduction in MMDs over the first month and of those, 81.1% continued to experience that level of response through 3 months. These analyses highlight several factors that may contribute to clinical decision making, as well as patient counseling regarding both trialing and stopping treatment.

Guidelines from the American Headache Society (AHS) outline parameters for giving a chosen preventive migraine treatment regimen an adequate trial before discontinuing. For injectable CGRP pathway–targeted monoclonal antibodies, it is recommended that clinical benefit be assessed after 3 months of a monthly treatment and after 6 months of a quarterly treatment [11]. For an oral generic preventive treatment, it is recommended that the medication be trialed for a minimum of 8 weeks at the target therapeutic dose before determining a lack of effectiveness [11]. Recent discussion of European guidelines by the European Headache Federation (EHF) suggested that, based on excellent tolerability data, if there is no other reason to stop treatment, the use of CGRP-targeted monoclonal antibodies should only be stopped when MMDs decrease to 4 or less; however, further studies are needed to investigate the long-term effects of discontinuation [23]. Although neither guideline directly references adequate trial or discontinuation recommendations for oral small-molecule CGRP receptor antagonists (gepants) [11, 24], both emphasize the improved tolerability of CGRP-targeted preventive migraine therapies over traditional nonspecific oral therapies (e.g., beta-blockers, calcium-channel blockers, and anti-depressants) [11, 23, 25]. Moreover, the most recently updated AHS position statement now recommends CGRP-targeted therapies, including gepants, as first-line treatment options for the preventive treatment of migraine, stating that the cumulative evidence for their efficacy, safety, and tolerability is significantly greater than that for any established migraine preventive therapy [25]. Our present data demonstrate both subsequent and sustained responses associated with atogepant, suggesting that sustained clinical benefits may be achieved with continued use of atogepant.

Considerations for long-term migraine treatment include potential delayed treatment effect, sustained treatment effect over 1 year with minimal diminishing, and dose-dependent effect. Findings suggest that those with migraine may benefit from atogepant treatment even if they do not have a response in the first month. Among ADVANCE study participants who did not experience a ≥50% reduction from baseline in MMDs with atogepant 60 mg in the first month, 61.4% proceeded to experience at least a 50% reduction in MMDs in month 2 or 3. In the LTS study, 62.4% experienced at least a 50% reduction in MHDs over the first quarter and 92.4% continued to experience that level of response through the second quarter. Additionally, in the LTS trial, over 90% of participants who experienced a ≥75% or 100% initial response had a ≥50% response in each subsequent quarter. Furthermore, relatively few participants who experienced a ≥50% initial response experienced < 25% response by the end of the ADVANCE (5.3–5.5%) or LTS (2.3%) trial. Results from the ADVANCE trial also suggest dose response effects on sustained benefits of atogepant treatment. At each level of response (≥50%, ≥75%, and 100%), the proportions of participants who had sustained response were highest in the groups that received atogepant 60 mg (Table 1).

Our post hoc analyses build on earlier findings and demonstrate that when an initial response to atogepant is experienced, these benefits are often sustained with continued treatment. In clinical trials and in practice, a ≥50% reduction from baseline in MMDs is considered to be a clinically meaningful response to preventive treatment for episodic migraine [26]. In a pivotal trial for atogepant, 52–62% of participants who received atogepant experienced ≥50% reduction in MMDs across the full 12-week double-blind treatment period [19]. A secondary analysis of ADVANCE demonstrated that a ≥50% reduction in MMDs with atogepant treatment occurred as early as the first 4 weeks and increased over time [27]. Furthermore, a separate analysis found that a significantly higher percentage of atogepant- vs. placebo-treated participants reported being migraine free on day 1 after treatment initiation (atogepant: 85.9–89.2%; placebo: 74.8%), reflecting a relatively rapid onset of action for atogepant [28]. Conventional oral preventive treatments often require titration periods to determine the appropriate dosage, whereas atogepant has demonstrated both early onset and sustained efficacy for the preventive treatment of migraine. Together, our findings may alter clinical decision making for the preventive treatment of migraine.

Analyses of injectable monoclonal antibodies that target the CGRP pathway for the treatment of EM have also evaluated duration of treatment responses [29, 30]. The STRIVE study demonstrated sustained efficacy in treating EM by showing that the majority of erenumab-treated participants who experienced a ≥50% reduction in MMDs during the last 3 months of the 24-week double-blind treatment period also had this response in the last 3 months of the 52-week dose-blind active treatment phase [29]. Results from the PROMISE-1 and PROMISE-2 studies demonstrated the sustained efficacy of intravenous eptinezumab through the analysis of cumulative months in which participants had a ≥50% reduction in MMDs [30, 31]. Additionally, a post hoc analysis of data from study participants with EM who received galcanezumab biweekly showed sustained response over 3 months and delayed response in a portion of them who did not have a response at month 1 but did see a ≥50% reduction in MMDs in months 2 or 3 [32].

A potential limitation of these post hoc analyses is the lack of a placebo control group. The efficacy of atogepant compared with placebo has previously been established using change from baseline in MMDs and response rates [17]. Our goal here was to evaluate the persistence of response. Additionally, both ADVANCE and the LTS trial were conducted in individuals with EM, which may limit the generalizability of the results to those with chronic migraine. However, results from a post hoc analysis of the PROGRESS trial demonstrated that most participants with chronic migraine who experienced an initial ≥30% or ≥50% MMD response had sustained the response throughout the 12-week trial [33]. An additional limitation of this analysis is that the proportion of participants who experienced a delayed treatment response was evaluated only for the ADVANCE trial. These topics should be further explored in future analyses.

## Conclusion

In the ADVANCE trial, > 70% of participants who experienced an initial ≥50% response at month 1 continued to experience this response throughout the 12-week treatment period, and > 50% of participants who did not experience a ≥50% response in month 1 proceeded to experience a ≥50% response in months 2 and 3. In the LTS trial, approximately 85% of participants who experienced a ≥50% initial response at quarter 1 continued to experience this response throughout the 52-week trial. Additionally, of participants who experienced a ≥75% or 100% initial response, > 90% had a ≥50% response in subsequent quarters. The results of these sustained response analyses demonstrate that, in the subgroup who experience an initial treatment response with atogepant, the majority have a sustained response for up to a year. These findings may provide additional support for decision making in clinical practice.

## Data Availability

AbbVie is committed to responsible data sharing regarding the clinical trials we sponsor. This includes access to anonymized, individual, and trial-level data (analysis data sets), as well as other information (e.g., protocols, clinical study reports, or analysis plans), as long as the trials are not part of an ongoing or planned regulatory submission. This includes requests for clinical trial data for unlicensed products and indications.These clinical trial data can be requested by any qualified researchers who engage in rigorous, independent, scientific research, and will be provided following review and approval of a research proposal, Statistical Analysis Plan (SAP), and execution of a Data Sharing Agreement (DSA). Data requests can be submitted at any time after approval in the US and Europe and after acceptance of this manuscript for publication. The data will be accessible for 12 months, with possible extensions considered. For more information on the process or to submit a request, visit the following link: https://vivli.org/ourmember/abbvie/ then select “Home.”

## Declarations

### Data sharing statement

AbbVie is committed to responsible data sharing regarding the clinical trials we sponsor. This includes access to anonymized, individual, and trial-level data (analysis data sets), as well as other information (e.g., protocols, clinical study reports, or analysis plans), as long as the trials are not part of an ongoing or planned regulatory submission. This includes requests for clinical trial data for unlicensed products and indications.

These clinical trial data can be requested by any qualified researchers who engage in rigorous, independent, scientific research, and will be provided following review and approval of a research proposal, Statistical Analysis Plan (SAP), and execution of a Data Sharing Agreement (DSA). Data requests can be submitted at any time after approval in the US and Europe and after acceptance of this manuscript for publication. The data will be accessible for 12 months, with possible extensions considered. For more information on the process or to submit a request, visit the following link: https://vivli.org/ourmember/abbvie/ then select “Home.”

## Abbreviations

AHS: American Headache Society
CGRP: Calcitonin gene–related peptide
EM: Episodic migraine
CM: Chronic migraine
LTS: Long-term safety
mITT: Modified intent-to-treat
MMD: Monthly migraine day
SC: Standard care
